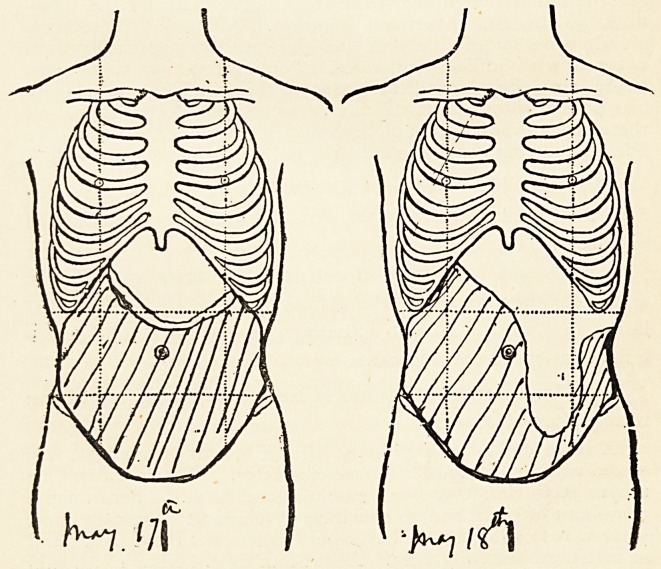# The Vagaries of Abdominal Tuberculosis: A Clinical Study

**Published:** 1904-03

**Authors:** James Swain

**Affiliations:** Professor of Surgery at University College, Bristol; Surgeon to the Bristol Royal Infirmary


### THE VAGARIES OF ABDOMINAL TUBERCULOSIS: A CLINICAL STUDY.

BY

JAMES SWAIN, M.S., M.D. Lond., F.R.C.S.,

*Professor of Surgery at University College, Bristol; Surgeon to the Bristol Royal Infirmary.*

---

THE diagnosis of abdominal tuberculosis is often beset with much difficulty, owing to the wide range of variability presented by the clinical symptoms. Indeed, the operative treatment of these cases may be said to date from the time when Sir Spencer Wells mistook a case of tuberculous peritonitis for an ovarian cyst: he closed the abdomen, and was astonished to find that the patient made a rapid recovery.

Since this classical error of 1862 we have learnt of many other conditions which abdominal tuberculosis may simulate, and I propose to relate a series of typical cases exemplifying the diagnostic difficulties which arise.

As in so much of abdominal surgery, our powers of diagnosis are coming on too slowly behind our capabilities of treatment; but this is partly compensated for by the fact that—as in Sir Spencer Wells's case—the exploratory incision frequently leads to a permanent cure of the condition. On the other hand, operation may hasten an inevitably fatal issue, and it is in these cases that we need more light in diagnosis to prevent falling into error.

Where medical treatment fails I believe that operation gives the best chance, if performed in about five weeks after an acute and five months after a chronic attack of primary abdominal tuberculosis, but of these straightforward cases I do not desire to speak. My object is to draw attention to the cases where the abnormality of the symptoms produces a difficulty in diagnosis over and above that which usually occurs.

**Case 1. Acute tuberculous peritonitis resembling intestinal obstruction and appendicitis.**

The patient, a female, aged 24 years, was suddenly seized with abdominal pain and vomiting after a heavy meal of beef and pudding. The pain was general at first, but subsequently was located in the right iliac fossa. The bowels acted after enemata only, and with difficulty. Five days after the commencement of the attack the vomit became stercoraceous. One sister died of phthisis, and the father was suffering from the same disease. The patient had an anxious expression, and the general condition was one of great gravity. The abdomen was much distended, tympanitic in front, dull in both flanks, but more on the right than the left side. There was general abdominal tenderness, but more marked in the right iliac fossa. On the fifth day of the attack an exploratory incision was made. On opening the peritoneum about twenty ounces of blood-stained fluid escaped. There was universal matting of the intestines, but the adhesions were easily separated in various directions, small quantities of fluid escaping from between the coils of gut. Both large and small intestines were of a bright red colour, being highly congested and swollen. On the parietal and visceral peritoneum were numerous discrete, elevated, milky-white, circular patches, varying in size from the head of a pin to that of a common tack. The vermiform appendix appeared normal. After operation the vomiting ceased, and two good motions were passed spontaneously; but the patient gradually sank, and died three days later.

Such an extremely sudden onset does occasionally occur in cases of acute tuberculous peritonitis, and the family history of phthisis might have led to a correct diagnosis; but in the face of a sudden onset, pain in the right iliac fossa, distended abdomen, and stercoraceous vomiting, the conclusion that we had to deal with a case of paralytic obstruction due to an appendicular peritonitis seemed irresistible.

These ultra-acute cases nearly always end in a general tuberculosis, and could we but diagnose them the surgeon would of course leave them severely alone; but the acuteness of the intestinal disturbance is usually so great that the majority of them are mistaken for intestinal obstruction, and a needless operation is performed.

The following extraordinarily complex case bears some relation to the one just recorded, and emphasises the great difficulty of diagnosis in a more marked degree.

**Case 2. Acute tuberculous peritonitis (? Addison's disease) resembling intestinal obstruction.**

The patient, a male, aged 48 years, had always been athletic and abstemious, but there was a previous history of hæmoptysis and fistula in ano, and a recent attack of what appeared to be appendicitis. After eating a dinner, at which he complained of having taken some "bad goose," he was seized at 3 a.m. on the morning following with marked purging and vomiting. This was regarded as being probably due to ptomaine poisoning, and at first there were no abdominal symptoms and no pain except when the bowels acted. While vomiting at 11 a.m. he suddenly fell back in a state of great collapse, and looked as if he were dying. After much energetic treatment he rallied a little, but the collapse did not pass off. At 3 p.m. a shifting dulness was noticed, first in the right flank and afterwards in both flanks. When I saw him about 6.30 p.m. he had an "earthy" look, and was much collapsed with a thready pulse. He was vomiting small quantities of mucus and altered blood. The abdomen was distended, and there was dulness in each flank, but there was no tenderness. Over the greater part of the abdomen there was hyper-resonance, the liver dulness was diminished, and the heart sounds were audible over the abdomen. I regarded the case as one of ruptured duodenal ulcer, and advised an exploratory incision; but before any operation could be commenced he vomited a "coffee-ground" material, collapsed, and rapidly died. A *post-mortem* examination was performed, and the doctor supplied me with the following account of it:—"There is no doubt in my mind that he died of Addison's disease. Both supra-renal bodies were involved beyond recognition in masses of caseous material, white as putty, intermingled with fibrous tissue. The spleen was twice its normal size, and the left end of the pancreas, the omentum, and the top of the left kidney (capsule and adrenal) were all fixed together by an irregular mass of caseous and fibrous substance the size of a medium-sized potato. This and the enlarged spleen produced the dulness in the left flank. The tubercular surrounding of the right adrenal was smaller. There was no free gas or fluid in the peritoneal cavity. There were signs of a perfectly healed appendicitis. Miliary tubercles were scattered over the bowel walls, but not visible elsewhere. A fibrous and caseous tubercular condition was present at the apex of each lung."

The writer of the above letter makes the following apposite comments on this case:—"Did you ever see a congeries of symptoms so exactly simulating a perforation or rupture of an abdominal organ with commencing peritonitis? . . . It is a question whether there really was ptomaine poisoning. If so it probably acted as a shock, and precipitated the final syncope of Addison's disease. Even the vomiting and diarrhœa may have done so."

Acute cases of Addison's disease and sudden death from syncope are well known, but I am not aware of any case of death under twenty-four hours without the existence of previous symptoms.

I am rather inclined to think that the simulation of acute abdominal mischief was more likely brought about by the acute miliary tuberculosis of the intestinal walls. This would establish a parallel condition to that found in Case 1, and yet allows of the existence of the pathological evidence of an Addison's disease which had not had time to declare itself clinically.

Whatever the true explanation may be, it does not relieve us much from the difficulty of diagnosis in so complicated a case.

We now come to the chronic form of abdominal tuberculosis. The cases in this division are attended with a similar degree of difficulty, but it is a fortunate fact that operation, though undertaken in error as to the exact condition, is attended by the most gratifying results.

**Case 3. Chronic tuberculous peritonitis resembling ovarian cyst.**

The patient, aged 41 years, had a period of ill-health, which was followed about six weeks before she came under observation by pain in the left side of the abdomen and a gradually increasing abdominal fulness, especially marked for the past fortnight. There was much emaciation, the patient weighing only a little over five stone. The lower abdomen was tense and prominent, and gave an indistinct sensation of fluctuation and thrill as high as two inches above the umbilicus. There was resonance above the pubes and in various parts over the front of the swelling. The temperature was 101°. On opening the abdomen, between the umbilicus and pubes, a large quantity of yellowish fluid (sp. gr. 1020, neutral) escaped. The intestines were much congested, and the parietal peritoneum was of a deep red colour. The surface of the intestines, uterus, etc., and the parietal peritoneum was very rough from innumerable deposits of miliary tubercle as far as the finger could reach. The intestines were matted together above and to the left, forming a sort of cavity by which the fluid was contained in the right iliac fossa, and thus giving rise to the resemblance of an ovarian cyst. The abdominal cavity was sponged out to remove the remains of the fluid, and the wound closed without flushing or drainage. Recovery was rapid, and six months after operation the patient weighed nearly seven stone, and was "better in health than she had been for a long time."

It is surprising how closely some of these cases of encysted peritonitis can resemble ovarian adeno-cystomata and parovarian cysts. The diagnosis is effected by observing the irregular relation of the areas of resonance and dulness (*e.g.*, resonance over the pubes is common in tuberculous peritonitis and rare in ovarian cysts), the presence of fever, and the rapid accumulation of the fluid. All these were present in the case reported above.

Another condition which helped me to correctly diagnose a similar case is the manner in which the relative positions of the dulness and resonance produced by the constantly changing amount of gaseous distension of the coils of intestine varies from day to day. This is exemplified by the accompanying diagrams, in which the dull area on two successive days is roughly represented by the shaded portion. It will, of course, be noticed that on each occasion the upper margin of the dull area was concave, which is the converse of that which obtains in the case of ovarian cystoma.

**Case 4. Chronic tuberculous peritonitis resembling appendicitis.**

A female, aged 6 years, had complained of marked pain in the right iliac fossa, especially before and during defecation, for four weeks. The bowels were loose, and the motions had been streaked with blood. The temperature kept at about 101°. Just internal to the right anterior superior iliac spine an indurated area could be felt reaching inwards for some 2½ inches from the iliac spine and shading off along Poupart's ligament. This swelling was fixed and tender. On examination, *per rectum*, some matting could be felt in the pelvis. An incision made near the right anterior iliac spine disclosed a large amount of tuberculous granulation tissue in the ileo-cæcal region, matting the intestines in the neighbourhood together. Numerous mesenteric glands were found enlarged, and the intestines generally were covered with miliary tubercles. The granulation tissue was freely scraped away and the abdomen closed. The temperature was normal after the fourth day from the time of operation, and there was a gradual gain of flesh and strength. After a visit to Weston, during convalescence, the child continued quite well.

The resemblance to appendicitis was very marked in this case, and it may be compared with the acute form mentioned in Case 1.

I have met with cases of a true tuberculous appendicitis, and even after operation I could not be perfectly sure that the lesion in Case 4 had not actually spread from a tuberculous inflammation of the appendix.

**Case 5. Tuberculous disease of mesenteric glands resembling retro-peritoneal abscess.**

A male child, aged 4 years, after a few days' malaise, was seized with vomiting of a severe character. This continued for twelve days, and there was much loss of flesh. There was no complaint of pain, and no elevation of temperature except once, when it rose to 99.4°. The bowels had acted throughout, and at first there was diarrhœa. About four days before I saw him there was a more or less defined tender swelling in the right hypochondrium for two days. Under chloroform the liver was felt to be somewhat enlarged, and this had probably caused the appearance of a "swelling." The child was listless and had a drawn look about the eyes. There was marked tenderness on pressure in the right hypochondrium, and an exploratory incision was decided on lest there should be an abscess. A 2-inch vertical incision was made in the right semilunar line, just below the costal margin. The small intestine was found to be congested. In the mesentery were numerous enlarged lymphatic glands, varying in size from a pea to a grape. These were yellowish-white in colour (? from early caseation) and of firm consistence. Nothing else abnormal was found, and the abdomen was closed. No sickness occurred after operation, and the patient rapidly put on flesh. In the course of six weeks the doctor wrote to say that the transformation was remarkable, and that the patient was "fat and chubby and overflowing with good spirits."

This was certainly a very happy termination to a most extraordinary case. It is unique in my experience; but if in the future it can be shown that operation does good in other cases of tuberculous mesenteric glands, it will bring this matter into line with the general effect of exploratory incision in cases of chronic abdominal tuberculosis.

**Case 6. Chronic tuberculous peritonitis resembling malignant disease of the stomach.**

A female, aged 45 years, complained of occasional "pain in the stomach" and sickness for about two years. The sickness generally came on soon after food, and the pain was relieved by the vomiting. For about a year she had noticed some swelling of the abdomen, and this had been more marked during the past five weeks, for which period she had been confined to bed, with an increased amount of pain and bilious vomit for the last fortnight. She had been getting thinner for twelve months, but more markedly so lately. Her eldest son was said to have "consumption." On examination the abdomen was found distended and resonant. Between the umbilicus and the right costal margin was a firm, irregular, more or less fixed mass about as large as the palm of the hand. This, in conjunction with the vomiting, suggested that it might be a tumour of the stomach. An examination of the gastric contents, however, showed a fair amount of acidity, and there was some matting in the pelvis. On opening the abdomen in the mid-line above the umbilicus, the tumour-like mass was found to consist of thickened, contracted, and adherent tuberculous omentum. There was matting of the intestines in the neighbourhood. The abdomen was closed, sickness ceased, and with the gradual improvement of the patient the tumour-like mass disappeared.

This case brings to mind those instances of "disappearing tumours," where masses indistinguishable clinically from malignant disease have disappeared spontaneously from the abdomen. It is highly probable that many such cases are really of a tuberculous nature.

The six examples just given show how difficult a matter it may be to correctly diagnose some cases of tuberculous peritonitis; and, in the five operations recorded, surgical measures were undertaken because of a wrong diagnosis or because the case was so obscure that an exploratory incision was required to make it clear.

In most of the cases the tuberculous nature of the mischief came as a welcome surprise, for on looking at the results obtained in all the operations, except that of the case of acute tuberculosis, it is clear that cœliotomy exercises a most beneficial effect even in most desperate cases. This fact fully justifies the resort to exploratory incision in all doubtful cases of tumour or inflammatory mischief in the peritoneal cavity. In the subacute and chronic forms of peritoneal tuberculosis operation, as we have seen, may be productive of much good; but in the acute miliary tuberculosis of the peritoneum, which resembles intestinal obstruction or appendicitis, operation is powerless to stop the fatal issue, and therefore should not be performed if it was possible to make a correct diagnosis.

It is in the acute cases that so much difficulty exists in diagnosis, and in the present state of our knowledge it is almost certain that some of them will continue to be mistaken for intestinal obstruction, but fortunately these cases are comparatively rare. Acute intestinal obstruction is such a fatal affection if left for more than twenty-four hours, that there is very little time for delay in cases where this condition is suspected, and it is better that we should occasionally operate by mistake on a case of acute miliary tuberculosis of the peritoneum than that an acute intestinal obstruction—which is much more common—should die without relief.

There was a family history of tubercle in both the acute cases, and attention to this fact in cases of apparently acute intestinal obstruction, where the obstruction does not appear to be absolute, may occasionally prevent us from falling into error.

In the chronic cases operation, though undertaken on a false diagnosis, is generally the best treatment for those instances of abdominal tuberculosis which resemble other diseases. The mistake therefore is not of much clinical importance; but in endeavouring to come to a right conclusion the following symptoms—most of which are exemplified in the cases related above—should be carefully observed: A tubercular history is generally present, and an elevation of temperature is not uncommon; in cases resembling an ovarian adeno-cystoma the accumulation of fluid is more rapid in tuberculosis than in ovarian cysts, there is resonance over the pubes, and the areas of resonance and dulness are irregularly disposed and vary from day to day; in other cases the long duration of the disease favours the diagnosis of tuberculosis, frequency of micturition is common, and matting of the pelvic viscera as felt on rectal examination (which should never be omitted) is often present.

## Figures and Tables

**Figure f1:**